# Low Density Lipoproteins as Circulating Fast Temperature Sensors

**DOI:** 10.1371/journal.pone.0004079

**Published:** 2008-12-30

**Authors:** Ruth Prassl, Magdalena Pregetter, Heinz Amenitsch, Manfred Kriechbaum, Robert Schwarzenbacher, John M. Chapman, Peter Laggner

**Affiliations:** 1 Institute of Biophysics and Nanosystems Research, Austrian Academy of Sciences, Graz, Austria; 2 Protein Engineering Biotechnology, University of Salzburg, Salzburg, Austria; 3 National Institute for Health and Medical Research (INSERM), Unite 551, Dyslipoproteinemia and Atherosclerosis Research Unit, Hôpital de la Pitié, Paris, France; Massachusetts Institute of Technology, United States of America

## Abstract

**Background:**

The potential physiological significance of the nanophase transition of neutral lipids in the core of low density lipoprotein (LDL) particles is dependent on whether the rate is fast enough to integrate small (±2°C) temperature changes in the blood circulation.

**Methodology/Principal Findings:**

Using sub-second, time-resolved small-angle X-ray scattering technology with synchrotron radiation, we have monitored the dynamics of structural changes within LDL, which were triggered by temperature-jumps and -drops, respectively. Our findings reveal that the melting transition is complete within less than 10 milliseconds. The freezing transition proceeds slowly with a half-time of approximately two seconds. Thus, the time period over which LDL particles reside in cooler regions of the body readily facilitates structural reorientation of the apolar core lipids.

**Conclusions/Significance:**

Low density lipoproteins, the biological nanoparticles responsible for the transport of cholesterol in blood, are shown to act as intrinsic nano-thermometers, which can follow the periodic temperature changes during blood circulation. Our results demonstrate that the lipid core in LDL changes from a liquid crystalline to an oily state within fractions of seconds. This may, through the coupling to the protein structure of LDL, have important repercussions on current theories of the role of LDL in the pathogenesis of atherosclerosis.

## Introduction

Circulating low density lipoproteins (LDL) are the main vehicles for cholesterol transport between tissues, sites of synthesis, utilisation and storage. The regulation of cholesterol uptake by endothelial cells through the non-atherogenic LDL receptor pathway or the pro-atherogenic scavenger receptor mechanism [1], [2] is critically dependent on the structure of LDL particles. Indeed, the structure and accessibility of apolipoprotein B-100 at the particle surface is of central relevance to specific ligand recognition in these regulatory processes [3], [4]. Apolipoprotein B100 structure has been shown to be highly sensitive to both chemical modification, such as oxidation [5], and to temperature change [6]. Thereby, the conformation of apolipoprotein B100 could be changed, which would affect its recognition by the LDL receptor as well as the receptor independent binding to cell surfaces [7], [8]. Furthermore, the structural state of LDL core lipids is intimately related to the dynamics of the intravascular metabolism of these cholesterol-rich particles [9], [10]. Thus, the susceptibility of LDL to oxidative modification, a triggering factor in the pathophysiological contribution of LDL to the development of atherosclerosis and cardiovascular disease, strongly depends on the physicochemical state of the core lipids [11].

Here, we report on the dynamics of structural changes within the core of LDL using time resolved small-angle X-ray scattering technology with synchrotron radiation. This technique allows to monitor phase transitions occurring within milliseconds. The earliest attempts to measure the kinetics of the phase transition in human LDL particles were conducted in 1979 by Mateu [12]. For technical reasons, the time resolution was limited to 15 seconds. Thus, with this very slow time-resolution, no conclusions regarding potentially relevant physiological kinetics could be drawn.

Since the original identification by Deckelbaum et al. [13] of a reversible temperature-induced transition process in LDL, it has been hypothesised that this transition may play a role in the progression of atherosclerosis through its effect on cellular pathways of LDL recognition and thus on LDL metabolism. Despite the fact that this lipid phase transition occurs slightly below the physiological temperature of blood in the abdomen [14], [15], and that the crystalline to isotropic melting transition of neutral lipids within LDL particles equally modulates the overall shape of the LDL particle [16], [17] and hence the conformation of apolipoprotein B100, no definitive analytical evidence has so far been available to support this hypothesis. It is therefore of paramount interest to evaluate the kinetics of such a transition in order to establish a potential pathophysiological link between the thermal LDL lipid transition and the atherosclerotic process.

**Figure 1:**
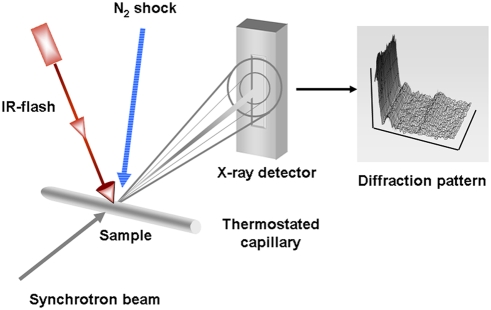
Sketch of the experimental setup. The experimental arrangement for time-resolved X-ray measurements at the Austrian SAXS–beamline at the ELETTRA synchrotron light source is shown. For T-jump experiments, an erbium laser beam (IR), wavelength λ = 1.5 µm, was directed via a prism onto the sample capillary which was thermostated with a Peltier unit. Laser pulse energy was 2 J within 2 ms resulting in an average T-jump amplitude of 10–12°C. The exposure time was 10 ms per frame. For T-drop experiments, the empty X-ray capillary was pre-cooled in a stream of nitrogen adjusted to −20°C. LDL samples, preheated to approx.10°C above the melting transition, were injected by a motor-driven syringe. A drop in temperature of about 20°C could be induced in about 3–4 s. The exposure time was 250 ms per frame.

The transition temperature of LDL varies significantly among different individuals, between extremes of 15 and 35°C. Such variation in temperature correlates closely with lipid composition, with higher transition temperatures in LDL particles with elevated cholesteryl ester to triglyceride ratios [14]. This range is lower than that typical of the body core temperature. However, in peripheral blood vessels such as the radial artery or the arteria dorsalis pedis [18], in finger tips and toe joints, or in the eyelids, blood temperature may drop to values below 30°C, which fall well within the range of the LDL transition. Such anatomical sites notably constitute loci for the preferential development of atheromata, i.e. the pathological deposition of cholesteryl esters [19]. Furthermore, in moderate and severe hypothermia, when the body can no longer maintain core temperature by peripheral vasoconstriction, then core temperature may drop below the LDL transition temperature [20], [21]. Thus, such loss of body temperature can permit transition of LDL core lipids towards a more rigid, ordered state. Moreover, the velocity of blood-flow can be as low as 0.3 mm/s in peripheral blood capillaries thereby favoring a residence time of several seconds for LDL particles in cooler regions of the body. With respect to the potential pathophysiological role of the LDL core lipid transition, then the key question concerns the intrinsic rate at which it can occur. Indeed, can LDL structure follow quasi-isothermal changes in blood temperature during its circulation, or does it remain adiabatically metastable in the molten-lipid state? The present report describes direct experimental investigations that should provide an unequivocal answer to this question. We have applied the same basic technique, i.e. small angle X-ray scattering (SAXS), as in the original study by Deckelbaum et al. [13], but in a fast, time resolving manner with the use of the high X-ray flux from a synchrotron source. Thus, we have been able to trigger the transition in either direction and to monitor associated structural changes in sub-second time intervals. Moreover, we designed specific instrumentation to evaluate the dynamics of T-jump and T-drop experiments in LDL, which is schematically depicted in Figure 1.

## Results and Discussion

**Figure 2:**
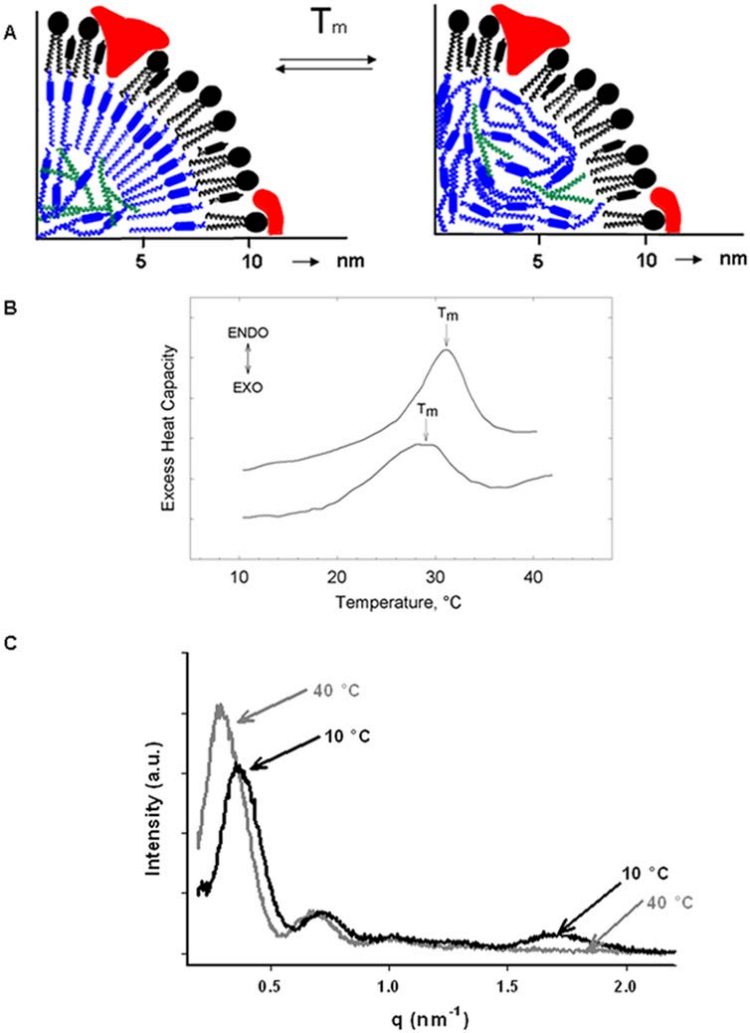
LDL nanophase transition. (A) Schematic of the thermotropic crystalline to isotropic transition in the apolar core lipids of LDL. Below Tm, the cholesteryl ester and triglyceride molecules (shown in blue and green, respectively) are arranged in two layers with a 3.6 nm repeat distance (left panel). Above Tm, the core lipids are in a fluid, oily state (right panel). The core lipids are surrounded by a monolayer of phospholipids and a single copy of apolipoprotein B100 (drawn in black and red, respectively). (B) Tm is typically determined by differential scanning calorimetry. The thermotropic transition is directly reflected in the small angle X-ray scattering curves. Static measurements performed at the SAXS – beamline at ELETTRA at 10°C and at 40°C are shown in (C). Upon heating above Tm, the intensity of the 1st side-maximum increases, and the 5th side-maximum vanishes. All scattering curves were recorded in the q-range of 0.09<q<2.30 nm-1 (q = 4π sin(θ)/λ, where 2θ is the scattering angle and λ the wavelength, λ = 0.15 nm).

Structural reorganisation of the core lipids in LDL takes place at the transition temperature T_m_ (Fig. 2A). T_m_ is typically determined by differential scanning calorimetry (Fig. 2B). However, the melting and freezing of the core lipids, i.e. of cholesteryl esters and triglycerides, can also be detected by distinct changes in SAXS patterns (Fig. 2C). At the melting transition, the intensity of the 1^st^ side-maximum in the scattering curve increases significantly in parallel to the total disappearance of the 5^th^ side-maximum at a scattering vector, q∼1.7 nm^−1^. Due to its low intensity, the latter maximum is highly sensitive to noise, rendering its analysis in time-resolved measurements difficult. Thus, for the T-jump experiments we selected the rise in intensity of the first side-maximum. In this way, we could achieve sufficiently precise data with a time resolution of 10 ms. With the chosen arrangement, we could obtain a temperature difference of approximately 10–12°C within the illuminated sample volume by one single laser flash [22], [23]. The starting temperature had to be chosen within the range corresponding to that of the crystalline phase of the LDL core. All experiments were performed on highly homogeneous distinct LDL subspecies [24].

**Figure 3:**
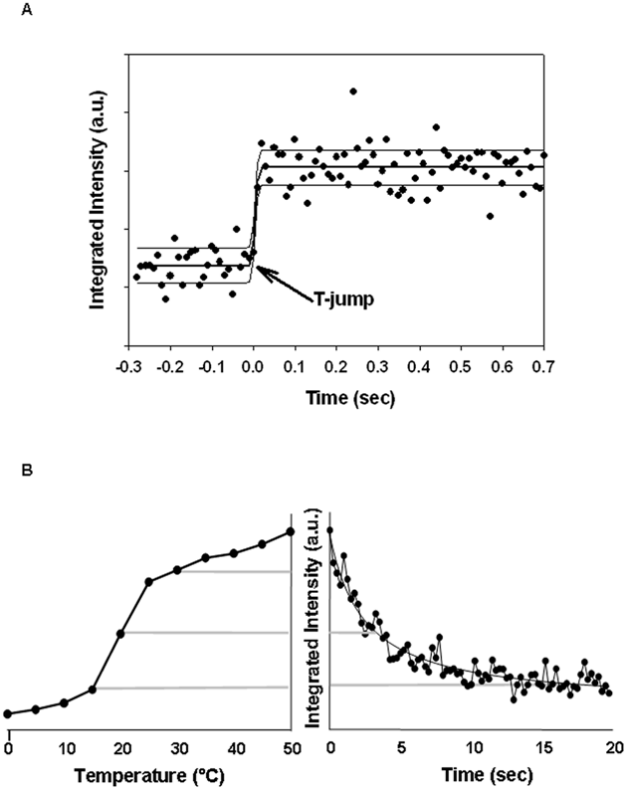
Time-resolved nanophase transition in LDL. The rise in the integrated intensity of the 1st side-maximum upon laser jump is shown as a function of time (A). The time slicing was 10 ms per image. The time point of laser flash is set to zero seconds. The error function of statistical variation displays a maximum inaccuracy in time of about 5 ms. Thus, the offset in transition is much shorter than the sampling time of 10 ms and the 2 ms of laser flash. The integrated intensities of the 1st side-maximum obtained by static measurements within a temperature range of 0°C and 50°C with a step width of 5°C (B, left panel) are correlated to the time-course of integrated intensities of the 1st side-maximum obtained by dynamic measurements (B, right panel). For static measurements, a measuring time of 30 s and an equilibration time of 10 minutes at each temperature was chosen. For dynamic measurements, the measuring time per frame was 250 ms. A half-time of 2 seconds, corresponding to a temperature drop of about 10°C, could be achieved to pass through the transition temperature. The decline in integrated intensity strictly followed the drop in temperature. Tm for the LDL sample shown was about 22°C, as determined by microcalorimetry.

The results of a T-jump experiment through the neutral lipid core phase transition of LDL are shown in Fig. 3A, and for better illustration, the changes in the integrated intensities of the first side maximum are depicted. The data for the first frame following the laser flash show the full height of intensity, which is characteristic of the molten state of the LDL core. These findings indicate that the phase transition takes place in a period of less than 10 ms. It is important to note that the time interval of 10 ms corresponds to the time-resolution of the measurement (10 ms per frame), and that this value is not necessarily the lowest limit of the transition rate.

As no method is available for radiative cooling in this temperature regime, we produced the sharp temperature drop with a pulsed cryo stream using N_2_ at 100 K. In this way, temperature drops of approx. 20°C could be achieved in 3–4 seconds. For calibration of the time/temperature scale, the integrated intensities of the 1^st^ side maximum of static measurements performed at different temperatures were correlated to the integrated intensities of the flash cooling experiment (Fig. 3B, left panel). Before the T-drop, the samples were equilibrated 10°C above the calorimetrically-determined T_m_ values. Thereafter the sample was injected; we subsequently observed a steep incline in the integrated intensities with a half-time of 2 seconds (Fig. 3B, right panel), which corresponds to the approximate time required for cooling of about 10°C. Based on this immediate intensity drop, we can infer that the rearrangement of core lipids proceeds within this time period. However, within the limits of this experiment, we could not achieve a higher cooling rate, and thus the freezing of the core lipids was limited by the cooling rate, while the intrinsic rate limit might actually be faster.

The apolar core lipids of LDL consist mainly of cholesteryl esters (40% or more of particle mass) and triglycerides (5% or less of particle mass), which show a well-defined internal layer-structure with a periodicity of about 3.6 nm below the melting temperature. These neutral lipids are arranged either as concentric shells [25]–[27] or, as more recently suggested by cryo-electronmicroscopy, as lamellae [16]. This lamellae-like layer organisation of LDL core lipids yields a new view of particle structure, which is consistent with low resolution X-ray diffraction patterns of LDL crystals [28], [29]. Indeed, this suggestion supports the notion that a nanophase separation occurs in LDL core lipids, in which triglyceride and cholesteryl ester molecules separate into distinct nanoenvironments [30]. However, irrespective of the internal molecular orientation of these apolar lipids, the distinct changes observed in our SAXS patterns are representative for the thermotropic transition of the LDL core.

This time-resolved study has revealed that the thermal-induced lipid re-organisation in LDL proceeds at the time scale of blood circulation. Such speed may be due to a low cooperativity of this process and may also be due to the disturbing influences of the minor lipophilic substances such as vitamin E and ubiquitous antioxidants, present in the LDL core. Clearly, no high activation energy is required for the phase transition, and measurements involving a time slicing of 10 ms or 250 ms were not indicative of any intermediate transition states. Since the rate of the “melting”-process is 10 ms or faster, and since the molecular rearrangement during “freezing” follows the sharp temperature-drop in the capillary, one can conclude that the transition takes place during the residence time of LDL in blood in appropriate regions of the human body. Thus, it is probable that LDL particles periodically undergo phase transitions as they pass the body core to peripheral vessels and return to the centre of the body under cold stress conditions. During this “frozen” period, not only the intravascular metabolism of LDL is affected but equally the activity of enzymes and lipid transfer proteins for which LDL is a substrate. Consequently, lipid transport triggered by specific transfer proteins such as cholesteryl ester transfer protein and enzymes such as lecithin-cholesterol acyltransferase may be reduced, probably as a result of the lower mobility and accessibility of lipids.

In addition to its occurrence in the natural circulation of blood, freezing of the LDL core may also be relevant to hypothermic cardiopulmonary bypass surgery, in which core body temperature may be reduced to 32°C for several hours [31]. During this time period, LDL particles might remain in their ordered state, depending on the respective lipid profile of the patient, which determines the actual core lipid transition temperature. Beyond these considerations, the “freezing-out” of the LDL core into microcompartments also implies that distinct local molecular environments may be created, in which certain active substances such as vitamins or drugs may accumulate transiently, leading to enhanced local activities [30]. Indeed, our results suggest that the core of circulating LDL particles, as a consequence of variations in blood temperature or acute hypothermia, may undergo a periodic redistribution of lipophilic constituents within its core nanodomains.

In summary, our study demonstrates that the thermal LDL lipid phase transition occurs over a time and temperature range, which is physiologically relevant, thereby suggesting that it is a significant factor in regulating not only the metabolism but potentially also the pathogenesis of LDL in atherosclerotic diseases.

## Materials and Methods

### Preparation of LDL

Circulating LDL is composed of several discrete subclasses, which can be subfractionated on the basis of their particle size and density [24]. Analytically, such distinct LDL subspecies offer the advantage of a high degree of structural homogeneity and were therefore used for this study. LDL (d 1.019–1.063 g/ml) was initially isolated from human plasma of normolipidemic, healthy volunteers and fractionated on discontinuous isopycnic density gradients as described earlier [24]. The volunteers provided fully informed consent to participate in this study by signing a written consent form before giving blood for research purposes only, an approval is given from the Hospital Review Board. The subspecies used for this study was of hydrated density range 1.0244–1.0435 g/ml; up to 80% of LDL in normolipidemic human plasma typically fall within this density range.

LDL preparations were dialysed exhaustively in the dark at 4°C against phosphate-buffered saline, pH 7.4, i.e. 10 mM phosphate buffer (Na_2_HPO_4_/KH_2_PO_4_), 150 mM NaCl, 270 µM EDTA, and 50 mg/L gentamycin. LDL was stored in the dark at 4°C in an argon atmosphere. Before use, LDL was concentrated with Centricon 10 concentrators (Amicon, MA) to a final concentration of about 7 mg/mL protein. The protein concentration was measured by the bicinchoninic acid assay (BCA-assay, Pierce, Netherlands). Total and free cholesterol were determined with CHOD-PAP enzymatic test kits (Roche Diagnostics and Wako Chemicals, Germany, respectively). Cholesteryl ester content was calculated as (total cholesterol−free cholesterol)×1.67 taking into account the average molecular weight ratio of cholesteryl ester to free cholesterol.

### Experimental protocol for T-jump and T-drop experiments

Time-resolved X-ray measurements were carried out at the Austrian SAXS-beamline at the synchrotron light source ELETTRA (Trieste, Italy), by using a monochromatic (λ = 0.15 nm) and mirror-focused X-ray beam in combination with a one-dimensional linear position-sensitive detector (Gabriel type, Grenoble, France) and a suitable time-slicing data-collection system (HCI, Hecus X-ray Systems, Graz, Austria). Position calibration of the detector was performed using the diffraction pattern of dry rat tail tendon collagen (d-spacing = 65 nm) as a reference. The sample contained within a glass capillary (1 mm diameter Mark capillary, Hilgenberg, Germany) of 0.1 mm wall thickness was placed in a thermoelectrically controlled cuvette (K-HR, A. Paar, Graz, Austria). For T-jump experiments, the capillary was centred in the crosspoint of the X-ray beam and perpendicular to the laser beam. The laser beam was guided by mirrors onto the capillary and attention was paid that the illuminated sample volume was larger than that hit by the X-ray beam. The energy output of the erbium laser (wavelength λ = 1.5 µm), used as the heating source for the T-jump experiments, was 2 J within the duration of a single 2 ms laser pulse. The absorption coefficient of water at this wavelength is 6.5 cm^−1^ leading to a T-jump amplitude of approximately 12°C [23]. The exposure time was 10 ms per image. One experiment consisted of 30 cycles of T-jumps with a delay-time of 120 s between two consecutive cycles.

For the T-drop experiments, a different experimental set-up was necessary. The sample was preheated about 10°C above the actual transition temperature (T_m_) in the outer zone located at the end of the capillary in the thermoelectrically-controlled cuvette. A stream of liquid nitrogen (Oxford Cryo Systems, Oxford, UK) was directed onto the middle of the capillary. The temperature of the liquid nitrogen was set to −20°C. Again, attention was paid that the cooled region of the capillary was larger than that hit by the X-ray beam. The preheated probe was injected into the cooled part of the capillary by a motor-driven syringe. In this way, a drop of temperature of 20°C could be induced in about 3–4 s. With this experimental set-up, no cycling was possible. Therefore, in order to achieve resolvable images, the measuring time per frame was set to 250 ms. The experiment was repeated eight times with two distinct LDL preparations with T_m_ values of 22°C and 30°C.

As a reference, static measurements were performed in a temperature range from 1 to 50°C with a step width of 5 degrees. Static measurements at 10 and 40°C, were also performed before and after the T-jump and T-drop experiments in order to ensure that no radiation damage did not affect the scattering patterns.

### Differential scanning calorimetry

The isothermal phase transition temperature (Tm) of the LDL preparations was determined by differential scanning microcalorimetry (MicroCal VP-DSC, MicroCal, Inc., Northampton, MA, USA) with a scanning rate of 1°C/min. Samples (1–2 mg/mL total LDL cholesterol) were loaded into the calorimeter sample cell at room temperature and the reference cell was filled with equilibrium buffer. The cells were pressurized with nitrogen to 250 kPa. Heat capacity functions were obtained after baseline subtraction and normalization of experimental data, given in µW, to heat capacity units (J/° g of cholesteryl esters). Data were collected on heating runs from 1°C to 50°C. Each sample was scanned two or three times and kept at 1°C for approximately 30 min between runs. Tm was taken from the maxima in the heat capacity versus temperature curves.
